# IPT coverage and determinants of care coverage in Tanzania

**DOI:** 10.5588/pha.22.0018

**Published:** 2022-09-21

**Authors:** H. Manisha, W. Amani, A. Garrib, M. Senkoro, S. Mfinanga

**Affiliations:** 1 National Institute for Medical Research Muhimbili Centre, Dar es Salaam, Tanzania; 2 Tanzania Field Epidemiology and Laboratory Training Programme, Dar es Salaam, Tanzania; 3 Muhimbili University of Health and Allied Sciences, Dar es Salaam, Tanzania; 4 Liverpool School of Tropical Medicine, Liverpool, UK; 5 Alliance for Africa Health and Research, Dar es Salaam, Tanzania

**Keywords:** HIV/AIDs, tuberculosis, TB, Dar es Salaam

## Abstract

**BACKGROUND:**

TB is a major cause of mortality worldwide, with the highest risk in people living with HIV/AIDS (PLWHA). Isoniazid preventive therapy (IPT), in combination with antiretroviral therapy (ART), reduces the overall incidence and mortality from TB by up to 90% among PLWHA. Tanzania has limited published data on IPT coverage among PLWHA.

**OBJECTIVE:**

To investigate coverage and determinants of IPT among PLWHA receiving care in selected care and treatment clinics in Dar es Salaam, Tanzania.

**METHODS:**

An analytical cross-sectional design to study 31,480 HIV-positive adults. Proportions and comparisons were obtained using χ^2^ tests, while determinants for IPT were assessed using adjusted multivariable analysis.

**RESULTS:**

The IPT coverage among eligible PLWHA was generally low (28.9%), with increased coverage over time. The determinants for IPT coverage included age >36 years, having WHO Clinical Stages 1 and 2 compared to 3 and 4, and having normal weight, or being overweight and obesity compared to underweight.

**CONCLUSION:**

IPT coverage in Dar es Salaam is very low; individuals with minor HIV disease severity were more likely to initiate IPT. This shows a possible gap in the prescribing practices among healthcare providers. More efforts to ensure IPT coverage implementation in Dar es Salaam are required.

## Abstract (fr)

**CONTEXTE:**

La TB est une cause majeure de mortalité dans le monde, le risque étant le plus élevé chez les personnes vivant avec le VIH/SIDA (PLWHA). Le traitement préventif à l’isoniazide (TPI), associé au traitement antirétroviral (ART), réduit l’incidence globale et la mortalité de la TB jusqu’à 90% chez les PLWHA. La Tanzanie dispose de peu de données publiées sur la couverture du TPI chez les PLWHA.

**OBJECTIF:**

Étudier la couverture et les déterminants du TPI chez les PLWHA recevant des soins dans des cliniques de soins et de traitement sélectionnées à Dar es Salaam, en Tanzanie.

**MÉTHODES:**

Une conception analytique transversale pour étudier 31 480 adultes séropositifs. Les proportions et les comparaisons ont été obtenues à l’aide de tests χ^2^, tandis que les déterminants du TPI ont été évalués à l’aide d’une analyse multivariable ajustée.

**RÉSULTATS:**

La couverture du TPI parmi les PLWHA admissibles était généralement faible (28,9%), avec une augmentation de la couverture au fil du temps. Les déterminants de la couverture du TPI comprenaient l’âge >36 ans, les stades cliniques 1 et 2 de l’OMS par rapport aux stades 3 et 4, et un poids normal ou un surpoids et une obésité par rapport à un poids insuffisant.

**CONCLUSION:**

La couverture du TPI à Dar es Salaam est très faible ; les personnes dont la gravité de la maladie VIH était mineure étaient plus susceptibles d’initier un TPI. Cela montre une possible lacune dans les pratiques de prescription parmi les prestataires de soins de santé. Des efforts supplémentaires sont nécessaires pour assurer la mise en œuvre de la couverture TPI à Dar es Salaam.

TB accounts for more than 10 million new cases per year worldwide.^1^,^2^ Almost a quarter of the world’s population is said to have TB infection (LTBI). People with compromised immune systems, such as people living with HIV/AIDS (PLWHA), have a higher risk of progressing from TBI to active TB.^3^ TB is the leading cause of death among PLWHA, accounting for around one in three HIV-related deaths.^4^ Isoniazid preventive therapy (IPT) reduces the risk of developing TB by 60–70%, and the combination of IPT and antiretroviral treatment (ART) reduces the overall incidence and mortality from TB by up to 90% in PLWHA.^5^,^6^

There are approximately 1.7 million PLWHA in Tanzania, with about 1.3 million on ART. The Dar es Salaam Region contributes about 11% of PLWHA on ART in Tanzania. HIV/AIDS-related deaths in Tanzania are estimated at 27,000 deaths annually.^7^,^8^ Tanzania notifies about 133,000 TB cases and about 26,000 TB-related deaths annually. Dar es Salaam region contributes about 20% of all notified TB cases in Tanzania.^9^ The Tanzanian national guidelines for the management of HIV and AIDs emphasise the importance of including TB preventive therapy (TPT), which includes the use of IPT, as part of the care package for PLWHA.^10^–^12^

Reliable data on IPT coverage among PLWHA in Tanzania are currently limited. It is important to get reliable data on the prevalence of TB and coverage of IPT for the Dar es Salaam region and the whole country. In this study, we investigated the coverage and determinants of IPT among PLWHA receiving care in selected care and treatment clinics (CTCs) in Dar es Salaam, Tanzania, from 1 June 2014 to 31 May 2019 using record review methods.

## METHODS

### Study design and setting

We used an analytical cross-sectional design to study de-identified secondary data of adult HIV-positive clients enrolled in CTCs of 17 health facilities in Dar es Salaam. The study period was from 1 June 2014 to 31 May 2019. The selected sites covered all levels of health facilities in Tanzania and were part of the RESPOND-AFRICA (Research Partnership for the Control of Chronic Diseases in Africa) projects. The sites offered an environment for smooth data collection with fewer resources required. These included Mwananyamala Referral Hospital, Shree Hindu Mandal Hospital, Sinza Hospital, Kimara Health Centre, Tandale Health Centre, Mnazi Mmoja Hospital, Amana Referral Hospital, Buguruni Health Centre, Tabata A Dispensary, Temeke Referral Hospital, Mbagala Rangi Tatu Hospital, Kigamboni Health Centre, Yombo Vituka Health Centre, Bunju Health Centre, Mbezi Health Centre, Tambukareli Dispensary and Vingunguti Health Centre.

### Eligibility criteria

All HIV-positive adults (⩾18 years) enrolled in CTCs of the Dar es Salaam Region for the first time between 1 June 2014 to 31 May 2019 were included.

### Exclusion criteria

All HIV-positive adults diagnosed with TB, those on anti-TB medications, those with a history of hepatitis, or those transferred in from other clinics were excluded.

### Data collection and management

The study involved retrospective data collection. The data were abstracted from the HIV programme (CTC-2) electronic database into Microsoft Excel 2019 (MicroSoft, Redmond, WA, USA) and exported to Stata v14.1 (Stata Corp Inc, College Station, TX, USA) for data management and analysis. Data were checked for consistency, i.e., all observations not meeting eligibility criteria and duplicates were removed from the dataset.

The national guidelines for the management of HIV and AIDS implemented before 2018 proposed the following: daily isoniazid (INH) 300 mg for 6 months for PLWHA in care and treatment comprising one complete cycle of IPT. IPT should then be repeated after 2 years from the first dose of the last IPT cycle. The eligibility criteria for IPT/TPT included all HIV-positive individuals with no signs or symptoms suggestive of active TB. Patients who had active TB in the past 2 years from the time of screening were not considered for TPT.^11^,^13^ Although post-2018 guidelines for the management of HIV and AIDs recommended the administration of 6 months of daily INH 300 mg to complete one cycle of IPT in PLWHA in care and treatment, only one IPT cycle was administered in a patient’s life time, and no repeat cycle was needed. Eligibility criteria included HIV-positive individuals who screened negative for active TB with no history of TB treatment. PLWHA with a history of TB treatment who completed their TB treatment were to immediately receive TPT for 6 months.^12^ Exclusion criteria for IPT/TPT in all available guidelines included alcohol abuse, non-adherence to long-term treatment, current or past history of hepatitis or medical contra-indication to INH.^10^–^12^

Derived variables such as BMI were generated, and variables such as age and WHO Clinical Stage that were collected at baseline were categorized into groups. Health facilities were classified into low, moderate, and high volumes depending on the number of clients that received care in the entire study duration, i.e., low <1000, moderate 1000 to <2000 and high 2000+. Coding of variables was also done to ensure conformity with models.

### Sampling and sample size estimation

We observed records of all (31,480) HIV-positive patients who enrolled in care and treatment clinics in the Dar es Salaam Region between 1 June 2014, and 31 May 2019. We observed that the minimum time for ART initiation was one day with median time from enrolment to IPT intake 150 days (interquartile range 75–240). Among the 31,480 observations, 2,425 (7.7%) records were dropped because of duplication (*n* = 444), registration before or after the study period (*n* = 24), being HIV-negative (*n* = 2), death at the date of enrolment (*n* = 85), transfer at the date of enrolment (*n* = 169) and not meeting the eligibility criteria (*n* = 1,701). Of the remaining records, 2,915 (3.2%) were excluded as they were either diagnosed with TB or were on anti-TB medications during the study period. The process of obtaining the final sample is shown in the flow diagram (Figure).

**FIGURE:**
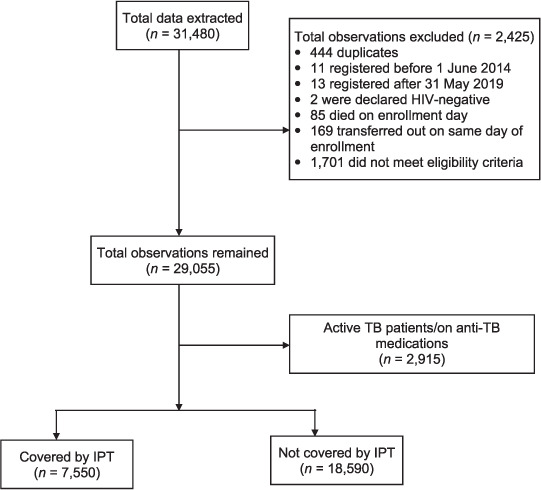
Data flow chart. IPT = isoniazid preventive therapy.

### Data analysis

The data were categorised into two time periods, 2014–2017 and 2018–2019, to reflect major changes in the national guidelines for the management of HIV/AIDS in Tanzania. The definition used for the purposes of this study was, “initiation of IPT within 12 months of enrolment”.

Proportions of IPT coverage were calculated and stratified by facility size, WHO Clinical Stage, patient category, CD4 count, age groups and body mass index (BMI). Factors for receiving IPT were also determined using adjusted multiple logistic regression analysis, odds ratios (ORs) with their respective 95% confidence intervals (CIs) were reported, and *P* < 0.05 was considered significant.

### Ethical considerations and informed consent

This protocol was submitted for ethical clearance to the National Health Research Ethics Committee (NatHREC) of the Tanzania Medical Research Coordinating Committee at the National Institute for Medical Research, Dar es Salaam, Tanzania (NIMR/HQ/R.8a/Vol.IX/3312). Consent was also obtained from administrative authorities of all the included clinics and health facility chiefs.

## RESULTS

### Baseline characteristics by IPT coverage

Overall IPT coverage during 12 months of enrolment was low, at about 28.9%, for eligible PLWHA for the entire study period from 2014 to 2019. We observed increased IPT coverage over time from 25.0% in 2014–2017 to 43.0% in 2018–2019.

IPT coverage was very low (23.5%) in the 18–24 years’ age group. This increased with age: respectively 27.3%, 31.5% and 32.2% in the 25–34, 35–44 and 45–55 years’ age groups. The 55–64 years’ age group experienced a decline in coverage to about 29.1%. The lowest coverage of 19.6% was observed in the >65 years’ age group.

IPT coverage increased with an increase in BMI, with 22.5% in the underweight, 29.1% in the normal weight, 31.4% in the overweight and 32.2% in the obese categories. The medium-volume health facilities showed higher coverage of IPT (31.8%) than low-volume (28.6%) and higher-volume (26.7%) facilities (Table 1).

**TABLE 1:** Baseline characteristics of study participants, 2014–2019

Characteristics	Year											

	2014–2017				2018–2019				Overall (2014–2019)			
		
	Total	IPT	No IPT	*P* value	Total	IPT	No IPT	*P* value	Total	IPT	No IPT	*P* value
Overall coverage	20,554	5,149 (25.0)	15,405 (75.0)	—	5,586	2,401 (43.0)	3,185 (57.0)	—	26,140	7,550 (28.9)	18,590 (71.1)	—
Sex												
Female	14,821	3,716 (25.1)	11,105 (74.9)	0.909	3,741	1,636 (43.7)	2,105 (56.3)	0.107	18,562	5,352 (28.8)	13,210 (71.2)	0.781
Male	5,733	1,433 (25.0)	4,300 (75.0)		1,845	765 (41.5)	1,080 (58.5)		7,578	2,198 (29.0)	5,380 (71.0)	
Age groups, years												
18–24	2,450	479 (19.6)	1,971 (80.4)	<0.001	705	262 (37.2)	443 (62.8)	<0.001	3,155	741 (23.5)	2,414 (76.5)	<0.001
25–34	7,167	1,698 (23.7)	5,469 (76.3)		1,919	782 (40.8)	1137 (59.2)		9,086	2,480 (27.3)	6,606 (72.7)	
35–44	6,599	1,802 (27.3)	4,797 (72.7)		1,783	839 (47.1)	944 (52.9)		8,382	2,641 (31.5)	5,741 (68.5)	
45–54	3,057	881 (28.8)	4,797 (71.2)		842	374 (44.4)	468 (55.6)		3,899	1,255 (32.2)	2,644 (67.8)	
55–64	959	239 (24.9)	720 (75.1)		256	115 (44.9)	141 (55.1)		1,215	354 (29.1)	861 (70.9)	
65+	322	50 (15.5)	272 (84.5)		81	29 (35.8)	52 (64.2)		403	70 (19.6)	324 (80.4)	
Binary age group, years												
18–35	10,393	2,387 (23.0)	8,006 (77.0)	<0.001	2,828	1,134 (40.1)	1,694 (59.9)	<0.001	13,221	3,521 (26.6)	9,700 (73.4)	<0.001
>36	10,161	2,762 (27.2)	7,399 (72.8)		2,760	1,267 (45.9)	1,491 (54.1)		12,919	4,029 (31.2)	8,890 (68.8)	
BMI, kg/m^2^												
<18.5 (under-weight)	2,754	563 (12.4)	2,191 (16.2)	<0.001	574	187 (32.6)	387 (67.4)	<0.001	3,328	750 (22.5)	2,578 (77.5)	<0.001
18.5–24.9 (normal weight)	9,839	2,524 (55.4)	7,315 (54.0)		2,723	1,135 (41.7)	1,588 (58.3)		12,562	3,659 (29.1)	8,903 (70.9)	
25.0–29.9 (overweight)	3,643	971 (21.3)	2,672 (19.7)		953	471 (49.4)	482 (50.6)		4,596	1,442 (31.4)	3,154 (68.6)	
>30 (obese)	1,854	494 (10.9)	1,360 (10.0)		573	287 (50.1)	286 (49.9)		2,427	781 (32.2)	1,646 (67.8)	
Size of health facility												
Low volume	4,724	1,172 (24.8)	3,552 (75.2)	0.001	1,431	590 (41.2)	841 (58.8)	<0.001	6,255	1,762 (28.6	4,393 (71.4)	<0.001
Medium volume	6,966	1,850 (26.6)	5,116 (73.4)		1,930	980 (50.8)	950 (49.2)		8,896	2,830 (31.8)	6,066 (68.2)	
High volume	8,874	2,132 (24.0)	6,742 (76.0)		2,218	827 (37.3)	1,391 (62.7)		11,092	2,959 (26.7)	8,133 (73.3)	

IPT = isoniazid preventive therapy; BMI = body mass index.

### IPT coverage and clinical features

In 2014–2019, patients with WHO Stages 1 and 2 had higher IPT coverage than those with WHO Stages 3 and 4 (31.4% vs. 24.4%; *P* < 0.001). The same groups had higher coverage in all study periods. In terms of CD4 count, those with CD4 ⩾200 cells/μL had slightly higher coverage (32.3%) than those with CD4 <200 cells/ μL (30.1%) in all study periods (Table 2).

**TABLE 2:** Association of IPT coverage with clinical HIV stage and CD4 count

Variable	2014–2016				2017–2019				Overall (2014–2019)			
		
	Total	IPT	No IPT	*P* value	Total	IPT	No IPT	*P* value	Total	IPT	No IPT	*P* value
WHO clinical stage												
Stage 1 and 2	12,229	3,291 (26.9)	8,938 (73.1)	<0.001	4,369	1,923 (44.0)	2,446 (56.0)	0.002	16,598	5,214 (31.4)	11,384 (68.6)	<0.001
Stage 3 and 4	8,295	1,854 (22.4)	6,441 (77.6)		1,202	467 (38.9)	733 (61.1)		9,544	2,321 (24.4)	7,174 (75.6)	
CD4 count at enrolment, cells/mm^3^												
<200	3,330	900 (27.0)	2,430 (73.0)	0.498	424	229 (54.0)	195 (46.0)	0.926	3,754	1,129 (30.1)	2,425 (69.9)	0.018
>200	5,986	1,657 (27.7)	4,329 (72.3)		1,253	680 (54.3)	573 (45.7)		7,239	2,337 (32.3)	4,902 (67.7)	

IPT = isoniazid preventive therapy.

### IPT coverage by health facilities

We observed heterogeneity across health facilities included in this study. The Kigamboni Health Centre had the highest IPT coverage (50.5%) within 12 months of enrolment during the entire study duration. while Bunju Health Centre had the lowest (15.3%) during the study entire study period (Table 3).

**TABLE 3:** IPT coverage by health facility, Tanzania

Facility	Total	IPT	No IPT	*P* value
Amana Referral Hospital	1892	647 (34.2)	1245 (65.8)	<0.001
Buguruni Health Centre	437	155 (45.5)	282 (64.5)	
Bunju Health Centre	844	129 (15.3)	715 (84.7)	
Kigamboni Health Centre	931	470 (50.5)	461 (49.5)	
Kimara Health Centre	645	136 (21.1)	509 (78.9)	
Mbagala Rangi Tatu Hospital	3972	895 (22.5)	3077 (77.5)	
Mbezi Health Centre	1518	493 (32.5)	10245 (67.5)	
Mnazi Mmoja Hospital	2273	1038 (45.7)	1235 (54.3)	
Mwananyamala Referral Hospital	3057	796 (26.0)	2261 (74.0)	
Shree Hindu Mandal Hospital	304	80 (26.3)	224 (73.7)	
Sinza Hospital	2172	622 (28.6)	1550 (71.4)	
Tabata A Dispensary	1691	323 (19.1)	1368 (80.9)	
Tambukareli Dispensary	1186	320 (27.0)	866 (73.0)	
Tandale Health Centre	1770	541 (30.6)	1229 (69.4)	
Temeke Referral Hospital	1648	439 (26.6)	1209 (73.4)	
Vingunguti Health Centre	1083	220 (20.3)	863 (79.7)	
Yombo Vituka Health	723	245 (33.9)	478 (66.1)	

IPT = isoniazid preventive therapy.

### Determinants of IPT coverage

PLWHA aged ⩾36 years were 1.37 (95% CI 1.25–1.50; *P* < 0.001) times more likely to have IPT coverage than those aged ⩽35 years after adjusting for other factors. PLWHA with WHO Clinical Stages 1 and 2 were 1.48 (95% CI 1.35–1.63; *P* < 0.001) times more likely to have IPT coverage than those with WHO clinical staging 3 and 4. Those with normal weight were 1.3 (95% CI 1.14–1.50; *P* < 0.001) times more likely to have IPT coverage than underweight patients, and overweight patients were 1.34 (95% CI 1.14–1.57; *P* < 0.001) times more likely to have coverage than underweight patients (Table 4).

**TABLE 4:** Multivariable analysis of factors for IPT coverage in CTCs in Dar es Salaam

Variables	Cumulative total						

	IPT	No IPT	*P* value	OR (95%CI)	*P* value	aOR (95%CI)	*P* value
Sex							
Female	5,352 (70.9)	13,210 (71.1)	0.781	Reference		Reference	
Male	2,198 (29.1)	5,380 (28.9)		1.01 (0.95–1.07)	0.781	0.94 (0.85–1.03)	0.191
Age, years							
18–35	3,521 (46.6)	9,700 (52.2)	<0.001	Reference		Reference	
⩾36	4,029 (53.4)	8,890 (47.8)		1.25 (1.18–1.32)	<0.001	1.37 (1.25–1.50)	<0.001
WHO HIV clinical stage							
Stage 1 and 2	5,214 (69.2)	11,384 (61.3)	<0.001	1.42 (1.34–1.50)	<0.001	1.48 (1.35–1.63)	<0.001
Stage 3 and 4	2,321 (30.8)	7,174 (38.7)		Reference		Reference	
CD4 count at enrolment, cells/mm3							
<200	1,129 (32.6)	2,425 (34.9)	0.018	Reference		Reference	
>200	2,337 (67.4)	4,902 (65.1)		1.11 (1.02–1.21)	0.018	1.04 (0.94–1.14)	0.466
BMI, kg/m^2^							
<18.5 (underweight)	750 (11.3)	2,578 (15.8)	<0.001	Reference		Reference	
18.5–24.9 (normal weight)	3,659 (55.2)	8,903 (54.7)		1.41 (1.29–1.55)	<0.001	1.30 (1.14–1.50)	<0.001
25.0–29.9 (overweight)	1,442 (21.7)	3,154 (19.4)		1.57 (1.42–1.74)	<0.001	1.34 (1.14–1.57)	<0.001
>30 (obese)	781 (11.9)	1,646 (10.1)		1.63 (1.45–1.74)	<0.001	1.17 (0.97–1.41)	0.101
Size of health facility							
Low volume	1,762 (28.6	4,393 (71.4)	<0.001	1.10 (1.02–1.18)	0.009	0.84 (0.74–0.96)	0.011
Medium volume.	2,830 (31.8)	6,066 (68.2)		1.28 (1.21–1.36)	<0.001	1.09 (0.99–1.19)	0.083
High volume	2,959 (26.7)	8,133 (73.3)		Reference			

IPT = isoniazid preventive therapy; CTC = care and treatment clinic; OR = odds ratio; CI = confidence interval; aOR = adjusted OR; BMI = body mass index.

## DISCUSSION

The IPT coverage in this study was generally low, with an overall coverage of 28.9% among 26,140 potentially eligible patients. The period 2014 to 2017 was important as the national guidelines recommended the administration of 6 months of daily INH 300 mg to complete one cycle of IPT, with the cycle repeated after 2 years.^11^,^14^ IPT coverage during this period was the lowest, with only 25% of eligible patients. IPT coverage during the period 2018–2019, when the guidelines recommended only one cycle of daily INH 300 mg for 6 months, was 43% of eligible patients. The increase in IPT coverage observed in these two study periods can be explained by the changes in policy guidelines that occurred between the two periods. These changes were accompanied by the promotion of the guidelines and improvements in supply chain which may have contributed to the observed increase.^10^,^12^ The low coverage of IPT in this study is similar to those reported in other countries in sub-Saharan Africa.^15^–^17^ The low IPT coverage has been attributed to several reasons, including interruptions in supply chains, and resulting in drug stockouts, ineffective TB screening, inefficient health service delivery, increasing pill burden, and inadequate high-level commitment and support for the IPT programme by programme managers and policymakers.^5^,^18^–^20^

The findings in this study showed increased IPT coverage with age, from age 18 to 54, and a decline in coverage in older patients of ⩾55 years. Also, multivariable analysis showed that those aged ⩾36 years were more likely to receive IPT than those aged <36 years. The findings of this study are not different from other studies in sub-Saharan Africa that reported higher IPT coverage among middle-aged patients.^21^,^22^ However, some studies have reported no association between IPT coverage and age.^23^,^24^ Relative high coverage among middle-aged patients reported in our study can be explained by the tendencies of young people to be more active in seeking healthcare than older patients who mostly depend on assistance from other people to access healthcare services, as shown in other studies.^25^,^26^

Having a normal weight (BMI 18.5–24.9 kg/m^2^) was significantly associated with higher coverage of IPT in the study population. In the multivariable analysis, we established that, participants with normal weight and those who were overweight were more likely to have IPT coverage than those with low BMI. This is similar to the findings of a study conducted in Tanzania showing an association between higher BMI and IPT initiation.^21^

We found that PLWHA with WHO Clinical Stages 1 and 2 had better IPT coverage than those with Clinical Stages 3 and 4. This finding is concurrent with other studies conducted in sub-Saharan Africa.^22^–^24^ Others have suggested that the low level of IPT coverage in WHO Clinical Stages 3 and 4 could be because clinicians are avoiding IPT in severely ill PLWHA due to the difficulty in ruling out TB disease in this population.^23^ This finding is alarming with regard to TB control, as PLWHA with WHO Clinical Stages 3 and 4 are at higher risk of developing TB. Other studies have reported that even with IPT provision, some PLWHA develop TB.^6^,^27^ The lack of IPT coverage among high-risk patients therefore undermines the efforts to control TB among PLWHA.

We observed variations in IPT coverage across health facilities such that coverage ranged from 15.3% at Bunju Heath Centre to 50.5% at Kigamboni Health Centre. Health facility volume tends to influence IPT stock requisitions. However, we found that medium-volume health facilities had higher IPT coverage than high-volume facilities, but this was not significant when adjusted for other factors. The observed variations may be due to difference in service delivery between medium-volume and high-volume health facilities. Other factors such as health facility ownership and location of the facilities may explain variations in IPT coverage across health facilities. The study findings are similar to another study that reported variation in IPT completion across health facilities.^17^

### Strengths and limitations

This is one of the few studies in Tanzania to report on IPT coverage using routine HIV programme data. This study also had a large number of patients to review from different sites. The use of HIV programme data from the CTC 2 database, which is updated daily, provided us with greater access to data and ensured improved data collection and more reliable findings. However, this study also had some limitations. Information on IPT used in this study did not include details on treatment completion and default or adherence levels during treatment.

### Generalisability

As this study focused only on selected health facilities using non-probability sampling techniques, results cannot be generalised. However, the selected facilities covered all levels of primary health care facilities in Tanzania, including district hospitals, health centres, dispensaries and not-for-profit health facilities.

## CONCLUSION AND RECOMMENDATIONS

IPT coverage among eligible PLWHA is low (28.9%), with individuals with lower HIV disease severity more likely to initiate IPT. However, PLWHA with advanced HIV or at higher WHO Clinical Stages are more vulnerable to TB. Our study shows a possible gap in the prescribing practices among healthcare providers. Access to TB diagnosis and diagnostic capability before IPT may also be an area for further investigation. More research is needed to further understand the facilitators of and barriers to implementing IPT coverage. In addition, policy interventions and peer influences may influence IPT coverage scale up among vulnerable groups in routine settings.
